# Electric fields unlock a transient vulnerability window for vancomycin-based treatment of staphylococcal biofilms

**DOI:** 10.1038/s41598-025-26053-6

**Published:** 2025-11-25

**Authors:** M. Balato, E. Roscetto, C. Petrarca, M. Vitelli, M. Aversa, U. Galdiero, M. R. Catania, L. Costanzo, S. M. Soto, M. Mariconda, G. Balato

**Affiliations:** 1 Department of Electrical Engineering and Information Technologies (DIETI), University of Naples Federico II, Via Claudio 21, Napoli (NA), 80125 Italy; 2 Department of Molecular Medicine and Medical Biotechnologies, University of Naples Federico II, Via Pansini 5, Napoli (NA), 80131 Italy; 3 Department of Engineering, Università degli Studi della Campania “Luigi Vanvitelli”, Via Roma, Aversa (CE), 81031 Italy; 4 Department of Public Health, University of Naples Federico II, Via Pansini 5, Napoli, 80131 NA Italy; 5 ISGlobal, Hospital Clínic - Universitat de Barcelona, Barcelona, Spain; 6 ISCIII - CIBER de Enfermedades Infecciosas, CIBERINFEC, Instituto de Salud Carlos III, Madrid, Spain

**Keywords:** Diseases, Microbiology

## Abstract

Bacterial biofilms pose a significant challenge to antibiotic treatment. Conventional therapeutic interventions often fail to effectively eradicate mature biofilms due to the protective extracellular matrix and altered physiological states of the embedded bacteria. In this study, we show that low-intensity sinusoidal electric fields induce a time-limited window of vulnerability in mature *Staphylococcus aureus* biofilms, increasing their susceptibility to vancomycin. In particular, the simultaneous application of electric fields and vancomycin during a transient vulnerability window results in a potentiated effect, leading to an approximately 3-log_10_ reduction in culturable cell counts relative to untreated controls. Conversely, the effectiveness of the antibiotic is reduced when vancomycin is administered after the cessation of the time-limited vulnerability window associated to the electrical stimulation, indicating a time-dependent reversibility of the electrical effect on the biofilm. These results suggest that the electric fields can create a critical phase of increased susceptibility to antibiotics, establishing a bioelectric strategy for biofilm control and highlighting the importance of treatment timing.

## Introduction

Biofilms, defined as complex communities of microorganisms encased within a self-produced extracellular matrix, have been identified as a major contributor to chronic and device-associated infections^1,2^. Their highly organised structure and altered metabolic states confer extraordinary tolerance to antimicrobial agents, often resulting in persistent infections that are recalcitrant to conventional antibiotic therapies^3^. Methicillin-resistant *Staphylococcus aureus* (MRSA) biofilms pose a severe clinical challenge, leading to prolonged hospitalization, increased healthcare costs, and elevated morbidity and mortality rates^4^. Despite the advances in antimicrobial development, the eradication of established biofilms remains a challenge, particularly because antibiotic penetration is impeded by the dense extracellular polymeric substance matrix, and sessile bacteria within biofilms are in dormant states that render them less susceptible to antibiotics targeting active cellular processes^5,6^. Consequently, there is a need for adjunctive strategies that can transiently disrupt biofilm integrity and enhance the efficacy of conventional antimicrobial treatments. Physical methods, including the application of electric fields, have emerged as a promising adjunct for the control of biofilms. Low-intensity electric fields have been shown to influence bacterial membrane potentials, increase permeability, and enhance the susceptibility of biofilm-associated bacteria to antibiotics^7,8^. The “bioelectric effect”, which was initially described as the enhancement of antibiotic action, has opened new avenues for the combination of physical and chemical treatments against biofilms^9,10^. However, the modes and temporal dynamics of this phenomenon remain poorly understood. This study aims to advance the understanding of the bioelectric effect and its role in modulating biofilm susceptibility to antibiotics. More specifically, the aim is to investigate whether the exposure of biofilms to low-intensity electric fields, which alone have a reversible disruptive effect, induces at least a transient vulnerability window during which biofilm-embedded bacteria become susceptible to even small doses of antibiotics, which alone have no detrimental effect. In practice, this study focuses on the critical role of treatment timing, i.e., whether antibiotics should be applied concurrently with or after electrical exposure, a question that has never been systematically addressed. Based on the findings of this study, it is hypothesised that low-intensity electric fields induce a transient and reversible phase of enhanced biofilm susceptibility, which is herein defined as the “Electric Field–Induced Vulnerability Window (EFIVW)”. Staphylococci, particularly S. *aureus*, are the predominant pathogens in surgical site and orthopedic biofilm-related infections (e.g., periprosthetic joint infections) and are associated with higher recurrence rates and poorer outcomes^11–13^. In this study, we considered a mature *S. aureus* biofilm as a model. Glycopeptide vancomycin was chosen as the antibiotic to be used in combination with the electric field as it is the main choice for treating MRSA infections. The mechanism of action of vancomycin is binding to the terminal d-Ala-d-Ala portion of the non-cross-linked cell wall intermediate Lipid II, blocking the extracellular assembly of peptidoglycan. Diffusion is believed to be the predominant force behind vancomycin penetration into biofilms^14^. However, its biofilm penetration ability is poor and therefore it is not effective in eradicating biofilm^15^. In this study the impact of synchronous and asynchronous applications of electrical stimulation and vancomycin is evaluated in detail. Indeed, synchronised treatment leads to significant biofilm reduction, whereas too much delayed antibiotic administration fails to fully exploit the induced vulnerability.

The results obtained establish a new conceptual framework for timing-optimised biofilm therapies and propose innovative strategies to overcome the resilience of pathogenic biofilms through the synergistic application of physical and chemical interventions. Moreover, the identification of the EFIVW provides a clinically actionable window for targeted biofilm disruption, with potential implications for perioperative protocols and the management of device-associated infections, particularly in orthopaedic and surgical contexts where biofilms are most recalcitrant.

## Methods

### Experimental setup

**Fig. 1:**
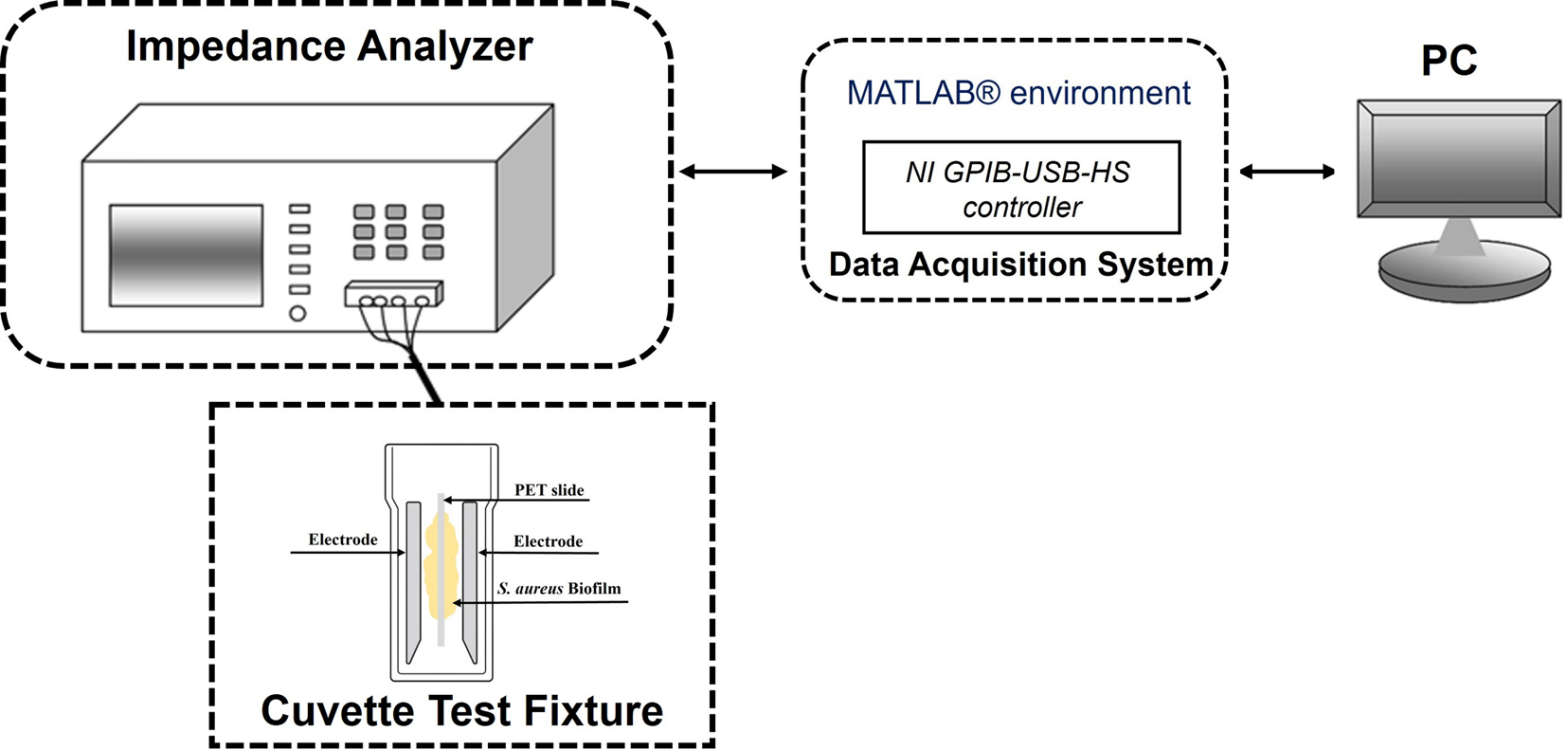
Schematic representation of the experimental setup.

An integrated platform was developed for the application of low-intensity sinusoidal electric fields to mature biofilms, as described in^16^. Briefly, the system consists of three core components: (i) a Cuvette Test Fixture, (ii) a Controlled Voltage Generator, and (iii) a Voltage Generator Controller (Fig. 1). In particular, the Cuvette Test Fixture (P1652088, Bio-Rad laboratories, Hercules, CA, USA) contains the biofilm under test and the electrodes for the application of a uniform electric field. The distance between the two electrodes was 0.4 cm. A 500 μm-thick PolyEthylene Terephthalate (PET) wafer was diced into 10 mm × 30 mm slides (width × length). Each PET slide was inserted into the cuvette in a vertical orientation, with the electrodes positioned laterally on either side of the slide, as illustrated in Fig. 1. This configuration provided a uniform environment suitable for biofilm growth. The electric field is applied by means of the Controlled Voltage Generator that imposes the voltage between the electrodes. The voltage applied by the generator is regulated by a Voltage Generator Controller, thereby producing a uniform sinusoidal electric field between the cuvette electrodes. The Root Mean Square (RMS) value of this field is 12.5 mV/cm, and the variable frequency is scanned logarithmically from 5 Hz to 13 MHz. Also, the time duration of the electric field generation can be properly controlled, and it is set, in the considered tests, to 4 and 6 min, as it is detailed in the following.

### Bacterial strain and growth conditions

This study was conducted using the MRSA reference strain ATCC 43,300. The strain was cultured in Brain–Heart Infusion (BHI) broth (Becton Dickinson Diagnostic Systems, Sparks, MD, USA) at 37 °C for 24 h. Aliquots were frozen at − 80 °C in BHI supplemented with 15% glycerol until further use. Identification and antibiotic susceptibility profile of the strain were performed using the automated VITEK2 system (bioMérieux, Marcy l’Étoile, France) and its proteomic profile was analysed by MALDI-TOF MS (Bruker Daltonics, Bremen, Germany).

### Biofilm formation assay on PET slides

*S. aureus* was first cultured on blood agar plates at 37 °C under aerobic conditions. After 19 h of incubation, the strain was resuspended in BHI broth to achieve a 0.5 McFarland standard suspension. Sterile PET slides (10 mm × 30 mm × 0.5 mm) were immersed in bacterial suspensions diluted 1:100 in fresh BHI medium supplemented with 1% glucose and incubated under static aerobic conditions at 37 °C. The culture medium was refreshed every day for five or six days.

### Experimental treatment conditions

Cuvettes containing the slides with *S. aureus* biofilm and 0.9% NaCl solution were divided into thirteen groups as it is detailed in Table 1. Groups 1 and 2 served as untreated control groups, consisting of 5-day-old (CT_5_) and 6-day-old (CT_6_) biofilms, respectively. Groups 3 and 4 included 5-day-old biofilms subjected to an Electric Treatment (ET) protocol to evaluate the effect of the electric field alone. These were compared to the 5-day-old untreated control. Specifically, Group 3 (ET_4_ protocol) and Group 4 (ET_6_ protocol) were exposed to a uniform sinusoidal electric field (RMS: 12.5 mV/cm; frequency range: 5 Hz to 13 MHz^16^ for 4 and 6 min, respectively (at the end of the 5-day period).

**Table 1:** Experimental groups and treatment conditions.

Group	Biofilm age	Electric treatment	Antibiotic treatment	Delay time	Overnight incubation	Abbreviation
1	5 days	ND	ND	ND	ND	CT_5_
2	6 days	ND	ND	ND	ND	CT_6_
3	5 days	4 min	ND	ND	ND	ET_4_
4	5 days	6 min	ND	ND	ND	ET_6_
5	5 days	6 min	ND	ND	YES	ET_6_-ON
6	5 days	6 min	ND	6 min	YES	ET_6_–DT_6_–ON
7	5 days	6 min	ND	12 min	YES	ET_6_–DT_12_–ON
8	5 days	6 min	ND	60 min	YES	ET_6_– DT_60_–ON
9	5 days	ND	YES	ND	ND	VAN
10	5 days	6 min	Sync	0 min	ND	SYNC
11	5 days	6 min	Async	6 min	ND	ASYNC-DT_6_
12	5 days	6 min	Async	12 min	ND	ASYNC-DT_12_
13	5 days	6 min	Async	60 min	ND	ASYNC-DT_60_

ND = Not Done; ET = Electric Treatment; VAN = Vancomycin; ON = OverNight; DT = Delay Time; Sync = Synchronous treatment; Async = Asynchronous treatment.

Groups 5, 6, 7, and 8 consisted of 5-day-old biofilms exposed to the ET6 protocol and then kept in saline solution at room temperature for delay times (DTs) of 0 min (ET_6_–ON), 6 min (ET_6_–DT_6_–ON), 12 min (ET_6_–DT_12_–ON), or 60 min (ET_6_–DT_60_–ON) before incubation. After each delay, the biofilms were incubated Over-Night (ON) at 37 °C in BHI to allow post-treatment regrowth.

Group 9 consists of 5-day-old biofilms treated with 50 µg/mL vancomycin (Fisiopharma Srl) alone for 24 h at 37 °C (VAN). Group 10 underwent a synchronous combined treatment (SYNC), involving the simultaneous application of the electric field (RMS: 12.5 mV/cm; frequency range: 5 Hz to 13 MHz^16^ and vancomycin exposure. These 5-day-old biofilms received a ET_6_ protocol in the presence of 50 µg/mL vancomycin (SYNC protocol), followed by ON incubation at 37 °C in BHI. Groups 11, 12, and 13 were designed to evaluate asynchronous treatment protocols, aimed at investigating the transient antibiotic susceptibility of biofilms induced by the ET_6_ protocol. In these groups, 5-day-old biofilms were first exposed to the ET_6_ protocol, followed by a delay period (DT) in saline at room temperature—6 min (ASYNC-DT_6_), 12 min (ASYNC-DT_12_), or 60 min (ASYNC-DT_60_). Following the delay, biofilms were treated with 50 µg/mL vancomycin for 24 h at 37 °C. Each experiment was performed in triplicate and repeated twice independently.

### Colony-forming unit (CFU) assay

The efficacy of the bioelectric treatment on *S. aureus* biofilms was evaluated by quantifying the bacterial load attributable solely to cultivable cells, expressed as CFU/mL. After treatments, control and test slides were gently washed with PBS to remove non-adherent cells. The remaining biofilms were detached and suspended by vortexing for 5 min using 0.1% dithiothreitol (DTT) solution^17^. Tenfold serial dilutions of the obtained suspensions were plated on Mueller–Hinton agar and, after 18–20 h at 37 °C, the resulting colonies were counted. CFU/mL values were expressed in exponential notation and as the percentage of cell death relative to the untreated control. To ensure complete removal of any remaining attached cells, the slides recovered after the DTT treatment were incubated in BHI overnight at 37 °C. Each experiment was performed in triplicate and repeated twice independently.

### Confocal laser scanning microscopy (CLSM) analysis

Biofilm viability was assessed using the LIVE/DEAD Bacterial Viability Kit (Life Technologies, Monza, Italy), which contains a combination of SYTO 9 (staining both live and dead bacteria in green) and propidium iodide PI (staining dead bacteria in red and quenching the green fluorescence). Images were acquired from three randomly selected areas per sample with a 20x objective lens, and sequential optical Sect. (1 μm) were collected along the z-axis to capture the complete biofilm structure.

### Statistical analysis

Experimental data are presented as mean values ± standard deviations. The antibiofilm activity of each treatment condition was evaluated by quantifying the reduction in colony-forming units (CFU/mL), which was expressed on a logarithmic scale. Each analysis was performed independently, as the experimental designs did not include crossed factors. The analysis considered four independent experimental factors. The first, “*Electrical Treatment exposure*”, involved three distinct exposure durations: 0 min (CT_5_), 4 min (ET_4_), and 6 min (ET_6_). In this framework, the untreated control (CT5) is explicitly interpreted as 0-minute electrical exposure. The second factor, “*Delay interval before incubation*”, referred to electric treatment (ET_6_) followed by different delay times (0 min, 6 min, 12 min and 60 min) between the end of the electric exposure and the onset of the incubation phase (ET_6_-ON, ET_6_–DT_6_–ON, ET_6_–DT_12_–ON, ET_6_–DT_60_–ON). The third factor, designed as “*Treatment Type*”, encompassed three conditions: vancomycin administered alone (VAN), Electric field alone followed by incubation phase (ET_6_-ON), and synchronous electric field combined with vancomycin (SYNC). Finally, the fourth factor, “*Delay interval before vancomycin application*”, corresponded to asynchronous protocols with varying delay times (0 min, 6 min, 12 min, and 60 min) between the electric field and antibiotic application (SYNC, ASYNC-DT_6_, ASYNC-DT_12_, ASYNC-DT_60_). Differences among groups were analysed using one-way analysis of variance (ANOVA) followed by Tukey’s post-hoc test. At the same time, pairwise comparisons with the untreated control (CT_6_) were further assessed using the t-test. Statistical significance was set at *p* < 0.05. All analyses were conducted using IBM SPSS Statistics for Windows, Version 23.0 (IBM Corp., Armonk, NY, USA).

## Results

### Antibiotic susceptibility profile of test strain

Following the identification of *S. aureus* ATCC 43,300 by MALDI-TOF MS (score > 2), antibiotic susceptibility profile was assayed with VITEK2 System and the results interpreted according to EUCAST guidelines^18^ (Table 2).

**Table 2:** Antibiotic susceptibility profile of MRSA 43,300.

Antibiotics	MRSA ATCC 43,300	
	**MIC/** ***Conc***	**SIR**
Fusidic acid	<=0,5	S
Ceftaroline	1	S
Ciprofloxacin	<=0,5	S
Clindamycin	> 1	R
Daptomycin	<=0,5	S
Erythromycin	> 2	R
Fosfomycin w/GP6	<=16	S
Gentamicin	> 4	R
Linezolid	1	S
Moxifloxacin	<=0,25	S
Mupirocin (high level)	<=256	S
Oxacillin	> 2	R
Penicillin G	> 0,25	R
Teicoplanin	<=0,5	S
Tetracycline	<=0,5	S
Tigecycline	<=0,25	S
Trimethoprim/sulfamethoxazole	<=1/19	S
Vancomycin	1	S

### Effect of electric field exposure on biofilm cells culturability

**Fig. 2:**
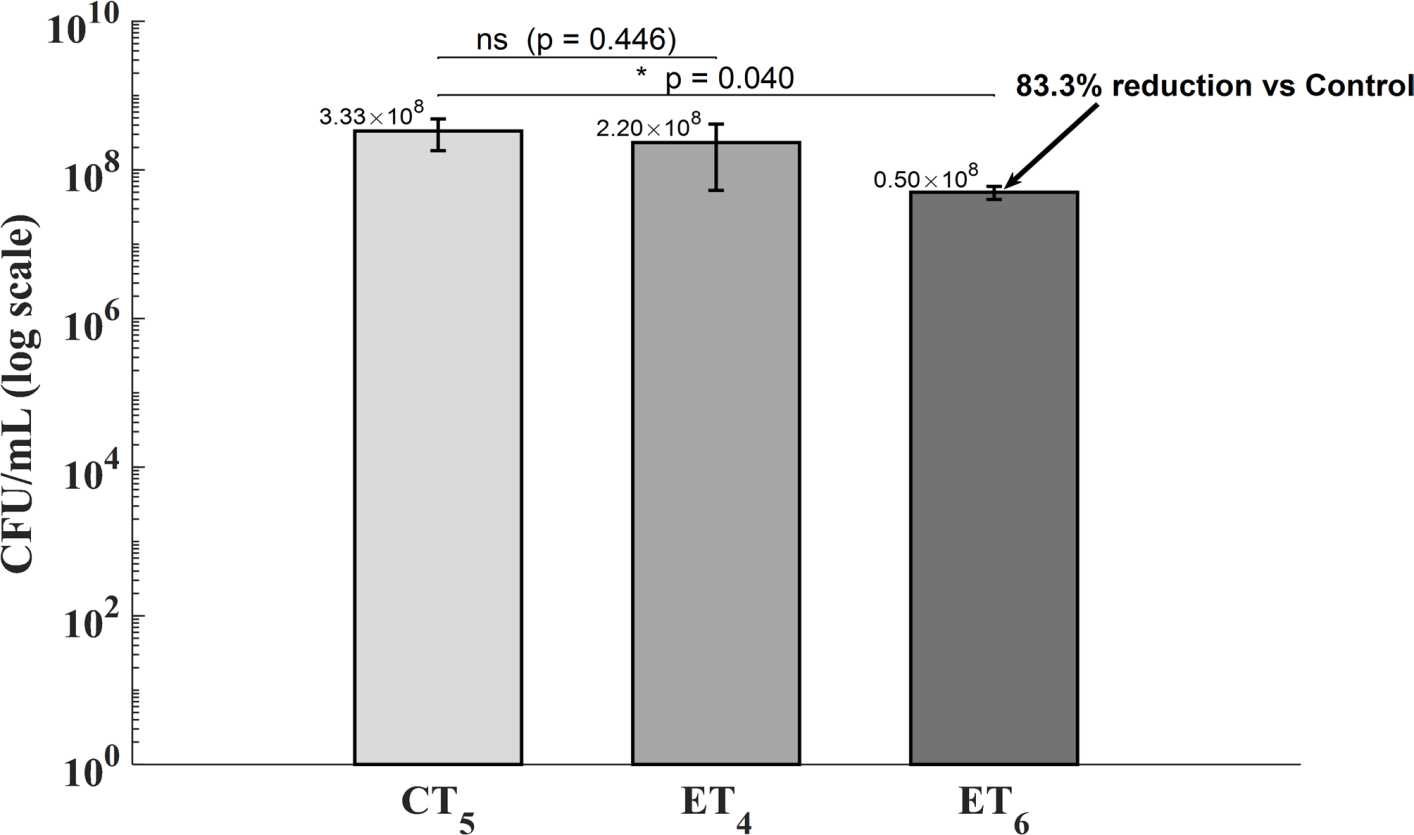
Colony-forming units (CFU/mL) in mature *Staphylococcus aureus* biofilms following electric field treatment at varying exposure times. Bars represent mean ± standard deviation (*n* = 3) of colony-forming unit counts (CFU/mL), shown on a logarithmic scale. Treatments included Electric Field exposure alone for 0 min (CT_5_), 4 min (ET_4_), and 6 min (ET_6_). Statistical significance was determined using one-way ANOVA followed by Tukey’s post-hoc test. A reduction of 83.3% was observed in ET_6_ compared to the control (*p* = 0.040). No significant difference was detected between CT_5_ and ET_4_ (*p* = 0.446).

The impact of electric field exposure was examined by comparing CFU counts across untreated controls (CT_5_), an ET_4_ protocol, and an ET_6_ protocol. As shown in Fig. 2, the electric field exhibited a time-dependent antibiofilm effect, as indicated by the differences observed among groups (*p* = 0.046). ET_6_ protocol significantly reduced culturable cells within the biofilm compared to CT_5_ (0.50 × 10⁸ ± 0.10 vs. 3.33 × 10⁸ ± 1.52 CFU/mL, *p* = 0.040), corresponding to an 83.3% reduction. Conversely, the ET_4_ protocol did not significantly differ from CT_5_ (*p* = 0.446), reflecting high variability and suggesting that a threshold duration is required for efficacy. These results underscore the importance of exposure time in optimising electric field-based interventions, as previously highlighted by the same authors in^16^. Based on these observations, subsequent analyses focused on the ET_6_ protocol, which has emerged as the most effective treatment condition within the current experimental framework.

### Post-electric treatment regrowth analysis

To assess the persistence of electric field–induced effects on biofilm culturability, the onset of incubation was delayed by 0 min (ET_6_-ON), 6 min (ET_6_–DT_6_–ON), 12 min (ET_6_–DT_12_–ON), or 60 min (ET_6_–DT_60_–ON) following a 6-minute electric exposure. Although CFU counts showed a trend toward recovery with increasing delay, one-way ANOVA detected no significant differences among groups (*p* = 0.565). Tukey’s post hoc comparison using ET6-ON as the reference corroborated this pattern, with not pairwise contrast reaching statistical significance. These results suggest that the reduction in culturability induced by electric field exposure is transient and may progressively diminish in the absence of immediate incubation. These findings further indicate that the biofilm is not permanently disrupted by electrical stimulation alone but can restore its structural and metabolic integrity once the stimulus is withdrawn.

**Fig. 3:**
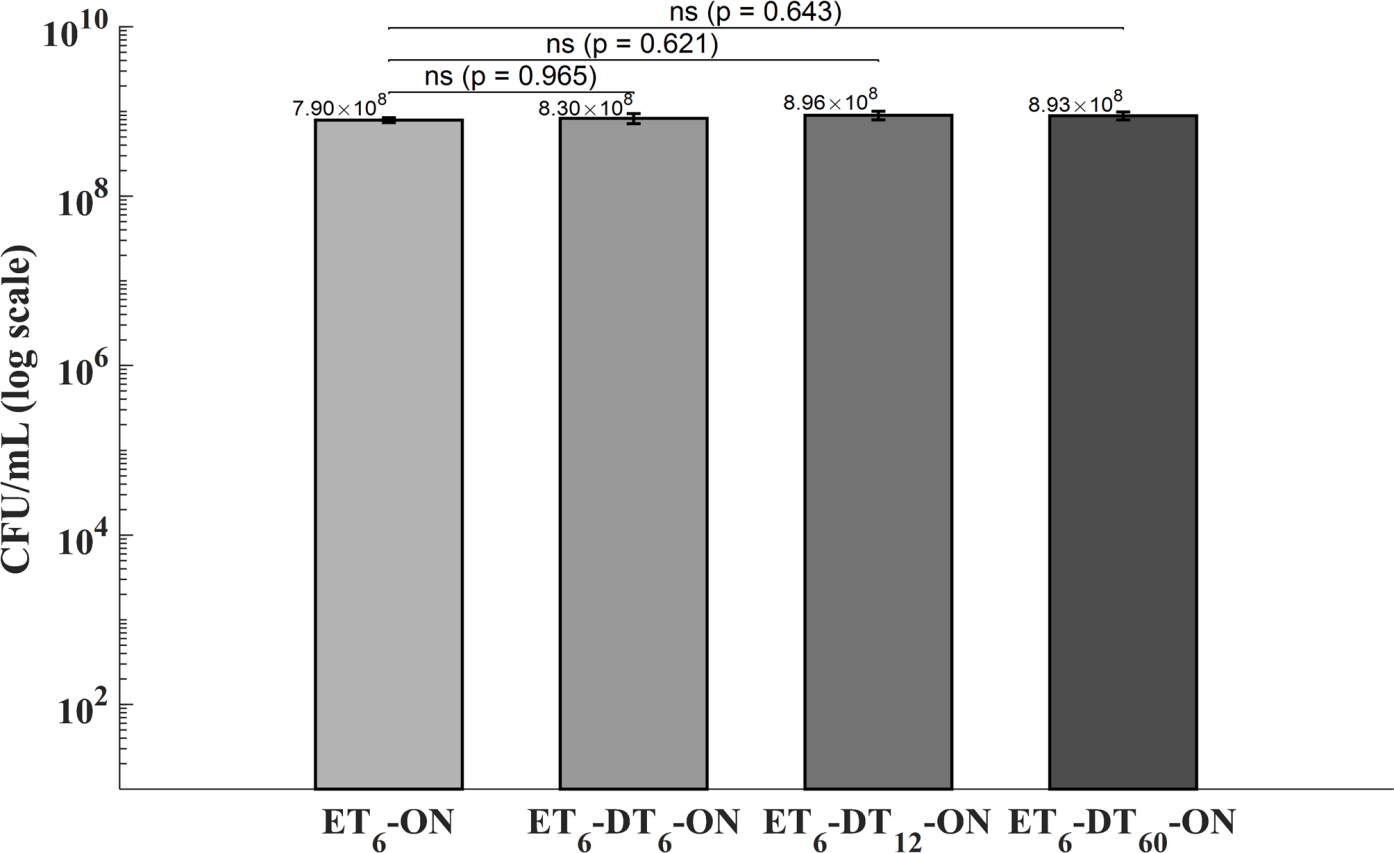
*Staphylococcus aureus* biofilms were exposed to a 6-minute electric field (ET_6_) and incubated after delay intervals of 0 min (ET_6_-ON), 6 min (ET_6_-DT_6_-ON), 12 min (ET_6_-DT_12_-ON), and 60 min (ET_6_-DT_60_-ON). Bars represent mean ± standard deviation (*n* = 3) of colony-forming unit counts (CFU/mL), shown on a logarithmic scale. One-way ANOVA detected no significant differences among groups (*p* = 0.565). Tukey’s post-hoc comparisons versus ET_6_-ON yielded *p* = 0.965 (ET_6_-ON vs. ET_6_-DT_6_-ON), *p* = 0.621 (ET_6_-ON vs. ET_6_-DT_12_-ON), and *p* = 0.643 (ET_6_-ON vs. ET_6_-DT_60_-ON), indicating a progressive but non-significant recovery of culturability over time. These results suggest that the electric-field effect on biofilm disruption is transient.

### Synergistic effect of synchronous electric field and vancomycin exposure

**Fig. 4:**
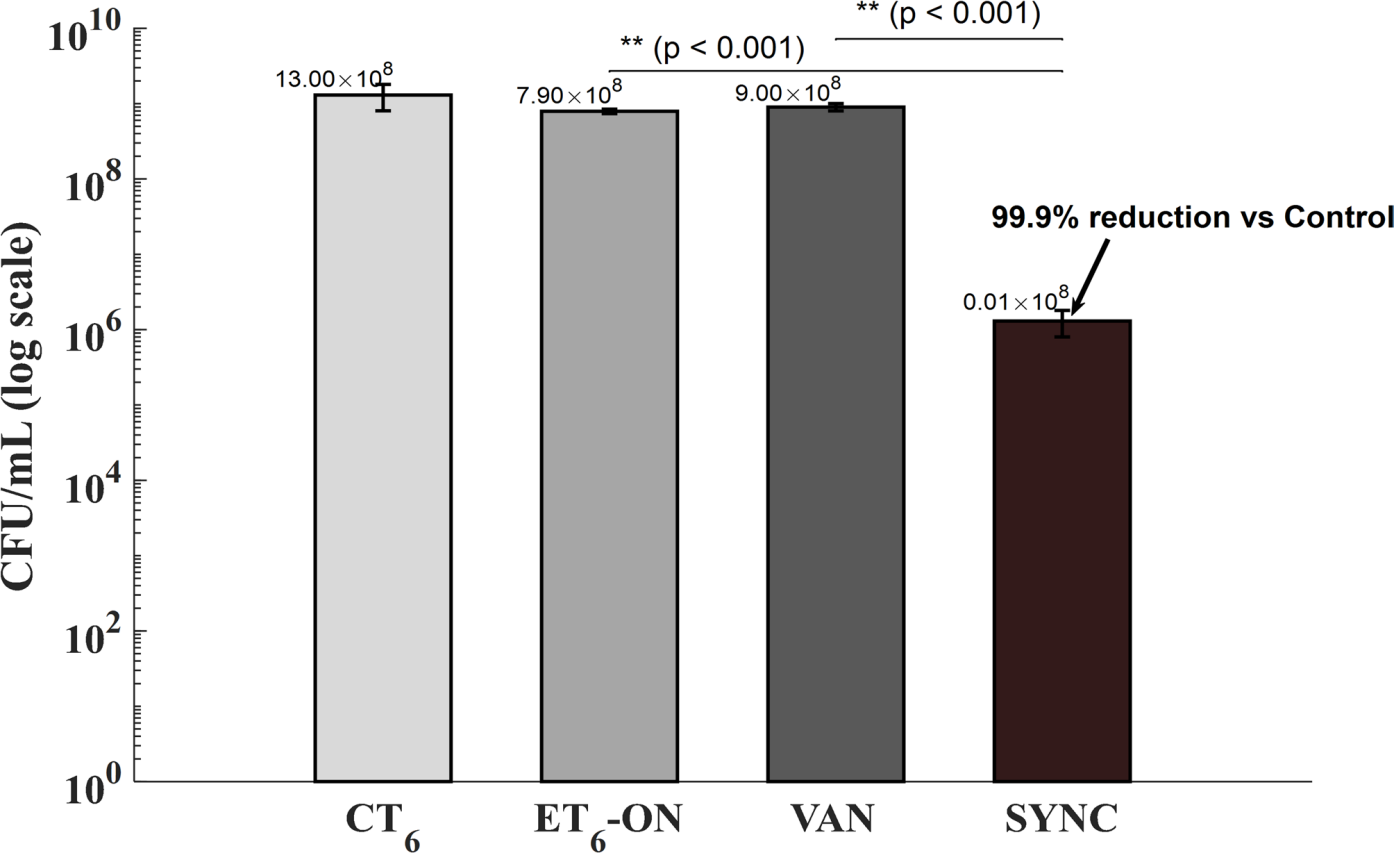
Colony-forming unit (CFU/mL) across different treatment conditions applied to mature *Staphylococcus aureus* biofilms. Bars represent mean ± standard deviation (*n* = 3) of colony-forming unit counts (CFU/mL), shown on a logarithmic scale. Three conditions were tested: biofilms exposed to electric field followed by overnight incubation (ET_6_-ON), biofilms treated with vancomycin alone (VAN), and biofilms subjected to synchronous electric field and vancomycin treatment (SYNC). Statistical analysis using one-way ANOVA revealed a highly significant difference across groups (*p* < 0.001). Tukey’s post-hoc comparisons showed that the SYNC protocol exhibited a statistically significant reduction compared to both VAN (*p* < 0.001) and ET_6_-ON (*p* < 0.001), confirming the synergistic effect of synchronised electric and antibiotic exposure. Pairwise *t*-test comparisons with the untreated control (CT6) revealed an approximately 3-log_10_ (~99.9%) reduction in CFU/mL relative to CT6 (*p* = 0.021).

Subsequent analysis evaluated the impact of synchronous exposure of biofilm to the electric field and vancomycin (SYNC protocol). A highly significant difference among treatment groups (*p* < 0.001) was observed (Fig. 4). The above protocol significantly outperformed both vancomycin alone (VAN protocol) (0.01 × 10⁸ ± 0.006 vs. 9.00 × 10⁸ ± 1.00 CFU/mL, *p <* 0.001) and the ET_6_-ON protocol (0.01 × 10⁸ ± 0.006 vs. 7.80 × 10⁸ ± 0.53 CFU/mL, *p* < 0.001). Pairwise *t*-test comparisons with the untreated control (CT6) revealed an approximately 3-log_10_ (~ 99.9%) reduction in culturable cells relative to CT6 (*p* = 0.021). These findings highlight a potent synergistic interaction between electric field exposure and antibiotic treatment when administered concurrently.

### Temporal limits of electric field–induced susceptibility

**Fig. 5:**
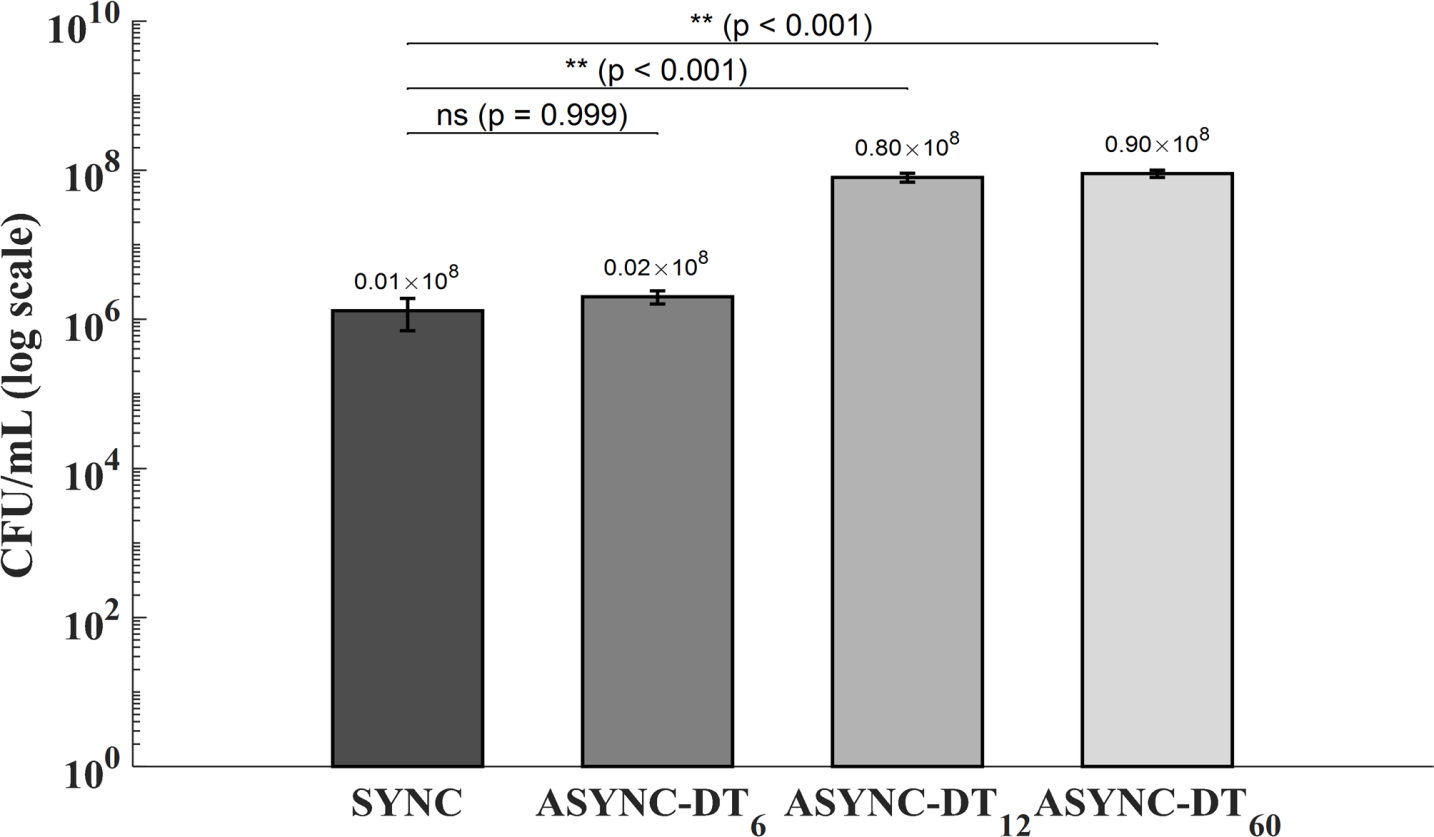
Colony-forming unit (CFU/mL) counts are shown for four treatment conditions: synchronous exposure to electric field and vancomycin (SYNC), and asynchronous protocols with a delay of 6 min (ASYNC-DT_6_), 12 min (ASYNC-DT_12_), and 60 min (ASYNC-DT_60_) in vancomycin exposure after electric field stimulation. Bars represent mean ± standard deviation (*n* = 3) of colony-forming unit counts (CFU/mL), shown on a logarithmic scale. One-way ANOVA revealed a highly significant difference across the groups (*p* < 0.001). Post-hoc comparisons confirmed that SYNC resulted in significantly lower CFU/mL than both ASYNC-DT_12_ (*p* < 0.001) and ASYNC-DT_60_ (*p* < 0.001), whereas no significant difference was observed between SYNC and ASYNC-DT_6_ (*p* = 0.999). These results support the existence of a transient EFIVW, maintained for up to 6 min post-stimulation, during which biofilm cells exhibit enhanced susceptibility to antibiotic treatment.

To assess the persistence of electric field–induced susceptibility, asynchronous protocols were evaluated (Fig. 5). ASYNC-DT_60_ protocol included a 60-minute delay between electrical and vancomycin exposure. Compared to the synchronous (SYNC) protocol, a statistically significant loss of efficacy was observed in ASYNC-DT_60_ protocol (*p* < 0.001), confirming that the antimicrobial effect was no longer present after 60 min. To further delineate the temporal boundaries of this effect (susceptibility window), SYNC protocol was also compared with ASYNC-DT_6_ and ASYNC-DT_12_ protocols, which involved 6-minute and 12-minute delays, respectively. No significant difference was observed between SYNC and ASYNC-DT_6_ protocols (*p* = 0.999), suggesting that the enhanced susceptibility was maintained for up to 6 min post-exposure. In contrast, ASYNC-DT_12_ differed significantly from SYNC (*p* < 0.001), indicating that the effect had substantially declined within 12 min. These findings support the existence of a transient EFIVW that lasts up to 6 min following stimulation, during which biofilm cells exhibit reversible susceptibility to antimicrobials. Importantly, this temporary state must be pharmacologically exploited within a narrow and defined timeframe to maximize therapeutic efficacy.

### Structural correlates observed by confocal imaging

**Fig. 6:**
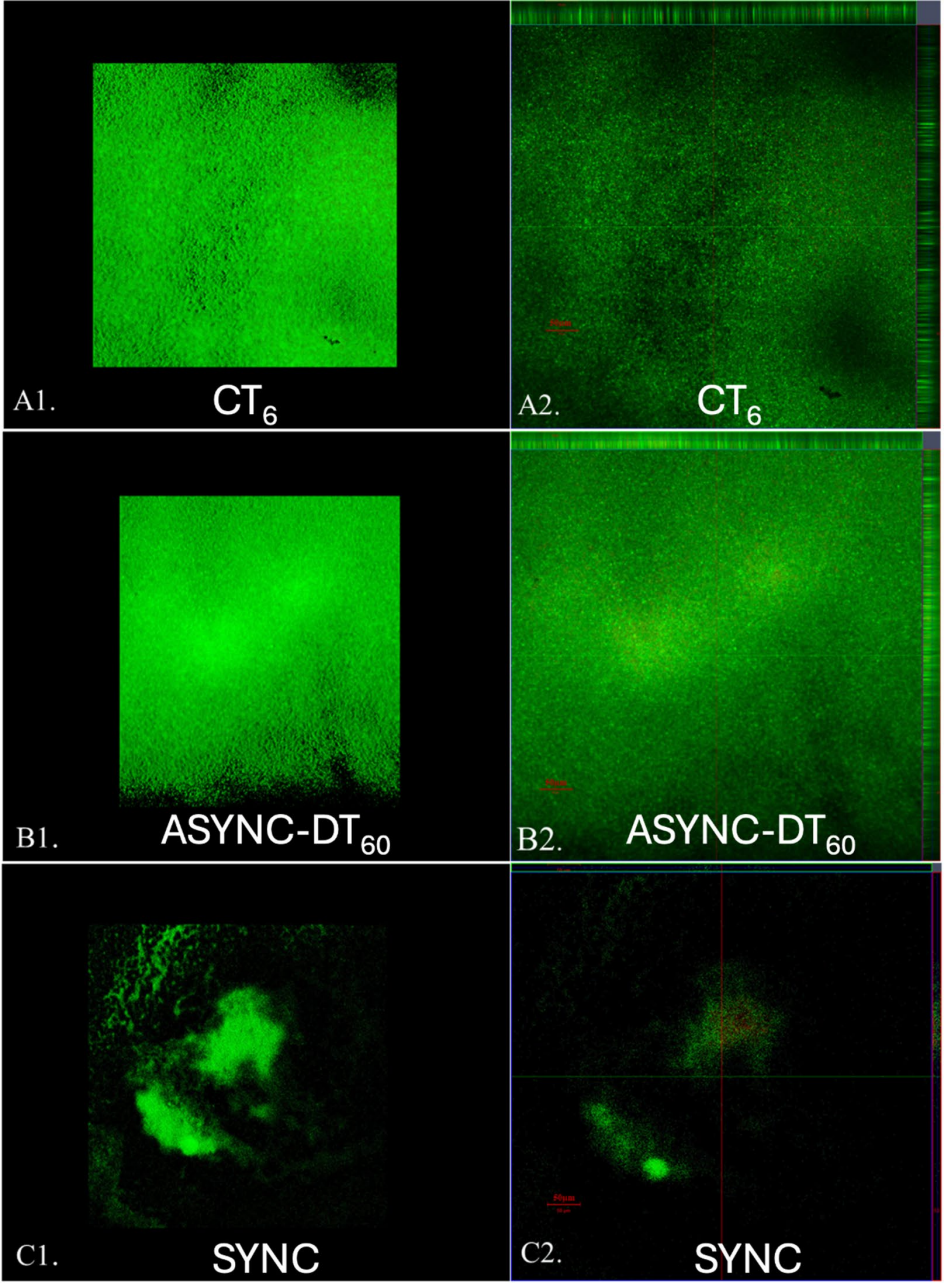
Visualization of S. aureus biofilm using Confocal Microscope: A1) 3D image in shadow format of a 6-day-old untreated biofilm (CT_6_); A2) Orthogonal Sect. (17/30) of CT_6_; B1) 3D image in shadow format of the biofilm exposed to ASYNC-DT_60_ treatment protocol; B2) Orthogonal Sect. (13/25) of ASYNC-DT_60_ treatment protocol; C1) 3D image in shadow format of the biofilm exposed to SYNC treatment protocol; C2) Orthogonal Sect. (4/11) of SYNC treatment protocol. Scale bars represent 50 μm as indicated in micrographs.

Confocal microscopy further corroborated these findings (Fig. 6). In the untreated control (CT_6_), a continuous biofilm layer of 30 μm (± 2) thickness was observed. In contrast, asynchronous treatment (ASYNC-DT_60_) produced partial biofilm thinning (23.3 ± 2 μm), whereas the synchronous protocol (SYNC) resulted in nearly complete biofilm detachment with few residual regions of lower density (11 μm ± 1). These structural differences visually reflect the quantitative reductions in CFU and highlight the mechanistic relevance of treatment timing.

## Discussion

### Overview of findings and comparison with previous studies

The present study demonstrates that low-intensity electric field (EF) exposure can transiently increase the susceptibility of mature *Staphylococcus aureus* biofilms to antibiotic treatment that, on its own, is insufficient to disrupt the biofilm. The SYNC treatment resulted in a synergistic effect, reducing culturable cells by more than 3-log_10_ compared with untreated controls. Our results align with earlier reports demonstrating enhanced antibiotic activity under electrical stimulation. For instance, Caubet et al.^19^ showed that both direct current (DC) and Radio Frequency Current (RFC) enhanced the efficacy of gentamicin and oxytetracycline against *E. coli* biofilms, with up to a 5-log₁₀ reduction under synchronous treatment. Zhao et al.^20^ similarly reported that brief, high-intensity electrical stimulation significantly enhanced vancomycin efficacy against in vivo MRSA biofilms, achieving a 2.6-log₁₀ reduction. However, their use of 75 mA/cm² raises safety concerns for clinical application. In contrast, the electrical parameters adopted in our study were several orders of magnitude lower. In contrast, the electrical parameters adopted in our study are several orders of magnitude lower. We refer to our stimulation as *low-intensity* because the applied field (≈ 12.5 mV/cm) and current density (< 20 µA/cm²) fall well within the microcurrent range, far below the levels known to induce irreversible electroporation or significant thermal effects. These mild electrical conditions are therefore sufficient to modulate biofilm physiology without causing safety issues. An applied voltage of 5 mV RMS across a 4 cm gap generated an electric field of approximately 12.5 mV/cm and currents ranging from 1 to 35 µA, corresponding to current densities between 0.5 and 17.5 µA/cm². These values lie within the microcurrent range (< 1 mA), which is generally recognized as safe and has been successfully used in medical and cosmetic applications^21,22^. Moreover, the applied conditions were well below the safety thresholds established by international standards (IEC 60601-2-10) and comparable to those used in FDA-approved medical devices^23–25^. Taken together, these considerations indicate that the electric fields employed in this study are both safe and biomedically relevant, while effectively modulating biofilm susceptibility. Kim et al.^26^ achieved a 6-log₁₀ reduction in *Escherichia coli* biofilms using 5.6 V/cm fields combined with gentamicin, while Hari et al.^27^ demonstrated enhanced doxycycline efficacy with a 2-minute exposure at 5 V and 11 mA in polymicrobial dental biofilms. However, the effectiveness of the antibiotic was significantly increased when used in combination with the electric field. Comparing our results with those of other Authors is difficult because the various studies used different experimental conditions. Electric field strength, treatment duration, bacterial strains, type and concentration of antibiotic, and maturity of biofilm are variables to be considered. Despite differences in bacterial species, antibiotics, and protocols, the trend is consistent: electric fields can potentiate antibiotic action if applied in a synchronous mode.

### Mechanistic insights: electric field effects on biofilm structure and antibiotic penetration

The observed effects can be attributed to electric field–induced alterations in biofilm structure and bacterial physiology. A body of research has indicated that electric fields have the capacity to enhance membrane permeability and compromise the integrity of the extracellular matrix^7,10^. Vancomycin and other glycopeptide antibiotics act by binding to the terminal D-alanyl-D-alanine dimer of the non-cross-linked cell wall precursor lipid II. This prevents the activity of penicillin-binding proteins, thereby stopping the precursor from being inserted into the bacterial peptidoglycan cell wall^28^. Since the target of vancomycin is extracellular, it can be assumed that the electric field may facilitate its diffusion through the biofilm matrix. Jefferson et al.^14^ demonstrated that vancomycin diffuses through the depths of biofilms at a significantly lower rate than in water. In their study, Zhao et al.^20^ demonstrated that applying a high-intensity current for 1 h increased both the penetration rate of vancomycin and the depth to which the drug penetrated wound tissues. In accordance with these observations, the results of this study support the hypothesis that electric field exposure generates a transient phase of biofilm destabilization. We can assume that, during this specific period, antibiotics are able to penetrate more effectively and exert a greater degree of bactericidal activity.

### The electric field–induced vulnerability window (EFIVW) as a novel concept

It is important to note that the destabilization of the biofilm by the electric field is only temporary. In fact, in instances where the administration of antibiotics was postponed, the biofilm appears to have restored at least some of its structural integrity, thereby diminishing the treatment’s effectiveness. Indeed, our study introduces the concept of the EFIVW, a transient and reversible phase during which biofilms become highly susceptible to antimicrobial agents. Although the enhancement of antibiotic efficacy by electric fields has been previously reported^10,19^, our findings advance this knowledge by demonstrating that treatment timing is not only influential but determinative, thereby introducing EFIVW as a clinically actionable target. The above concept may be mistakenly conflated with reversible biofilm electroporation^29^, as both involve transient increases in antimicrobial susceptibility following electric field exposure; however, while electroporation primarily affects membrane permeability at the single-cell level, EFIVW likely reflects a broader, time-dependent biofilm response involving electrochemical, metabolic, and structural perturbations.

### Synchronization and therapeutic timing: clinical implications

The substantial disparities observed between synchronous and asynchronous applications underscore the notion that the efficacy of treatment is contingent not solely on the existence of an electric field but also on the meticulous synchronization of treatment modalities. The clinical implications of these results are substantial. In the clinical management of Periprosthetic Joint Infections (PJI), mechanical, chemical, or electric debridement or electroceutical application is followed by systemic antibiotic administration. Such a sequence establishes an inherently asynchronous treatment paradigm, wherein antibiotics reach the infection site only after the initial biofilm-disruptive intervention. In this context, our observation that delayed antibiotic administration (as in ASYNC-DT_12_ and ASYNC-DT_60_) results in the loss of treatment efficacy closely mirrors the clinical challenge in PJIs, where systemic antibiotics often fail to reach the infected area within the optimal therapeutic window due to poor vascularization and the protective biofilm matrix. This parallel reinforces the translational relevance of the EFIVW, emphasizing that the therapeutic success of electroceutical interventions depends not only on the applied field parameters but also on the timing and synchronization of adjunctive antibiotic therapy.

### Translational potential and future directions

The ability to transiently disrupt these defenses using safe, low-intensity electric fields could enhance the efficacy of existing antibiotics and reduce the need for high-dose or prolonged therapies, potentially limiting adverse effects and resistance development. Notwithstanding the encouraging results of this study, it is imperative to acknowledge the limitations that must be considered when interpreting the findings. The experiments were conducted in a laboratory setting using a single bacterial strain. Future studies should explore the applicability of the EFIVW across different biofilm-forming species, polymicrobial communities, and clinically relevant models. Additionally, optimization of electric field parameters and exploration of combined physical and chemical interventions could further enhance treatment outcomes. The authors acknowledge that the role of charge distribution and its intimate relationship with field intensity may play a critical role in shaping the temporal dynamics of the EFIVW phenomenon. However, a systematic investigation of this aspect lies beyond the scope of the present study and will be addressed in future developments of this research. In conclusion, our results provide a new mechanistic insight into biofilm susceptibility modulation through electric field exposure. By elucidating the optimal timing for antibiotic administration, this study provides a foundation for the development of targeted therapeutic strategies against biofilm-associated infections. Ongoing investigations seek to extend these findings across diverse bacterial species.

## Conclusion

This study identifies and characterizes a previously unrecognised phenomenon: the Electric Field–Induced Vulnerability Window (EFIVW)—a transient, reversible state of heightened antibiotic susceptibility in mature *Staphylococcus aureus* biofilms. The findings demonstrate that the synchronous application of a low-intensity electric field and vancomycin results in a dramatic, reproducible reduction in cultivable biofilm cells, corresponding to nearly a 3-log_10_ reduction compared with untreated controls. A systematic evaluation of delayed treatment protocols (Asynchronous treatment protocols) has been undertaken, and it has been demonstrated that the enhanced susceptibility induced by the electric field is not permanent but instead persists for at least of 6 min following stimulation. Delaying antibiotic administration beyond this threshold – specifically to 12–60 min – results in a marked loss of therapeutic efficacy, confirming the time-sensitive nature of the EFIVW. These results establish precise temporal coordination as a critical parameter in the success of bioelectric–antibiotic combination therapies. Significantly, this research establishes a mechanistic and conceptual basis for future electroceutical strategies aimed at disrupting chronic biofilms, particularly in clinically challenging settings such as orthopedic implants and surgical site infections. Subsequent studies will seek to elucidate the molecular basis of the EFIVW and expand its application across a range of bacterial species and antimicrobial agents.

## Data Availability

All data generated or analysed during this study are included in this published article.

## Declarations

### Supplementary Information

Correspondence and requests for materials should be addressed to M.B and E.R.
